# Magnitude of Neonatal Sepsis and Factors Associated with It among Neonates Admitted to the Intensive Care Units of Neonate in the Primary Hospital of Hawzen, Tigray, Ethiopia, 2020

**DOI:** 10.1155/2024/7393056

**Published:** 2024-08-22

**Authors:** Fre Gebremeskel, Haftay Gebremedhin, Medhin Mehari

**Affiliations:** College of Medicine and Health Sciences Adigrat University, Adigrat, Tigray, Ethiopia

## Abstract

**Purpose:**

Neonatal sepsis contributes substantially to neonatal mortality and morbidity and is an ongoing major global public health problem particularly in developing countries. A significant proportion of mothers give birth in primary health care, but studies regarding neonatal sepsis and its associated factors among admitted neonates are limited to the hospital which may not be generalized to the primary health care unit. Therefore, this study aimed to assess the proportion of neonatal sepsis and associated factors in the study areas.

**Objective:**

To assess the magnitude of neonatal sepsis and its associated factors among neonates admitted to Neonatal Intensive Care Units (NICUs) of Hawzen Primary Hospital, Eastern Zone, Tigray, North Ethiopia, 2020.

**Methods:**

An institution-based cross-sectional study design was carried out among 290 study participants in Hawzen Primary Hospital in January–March/2020. A systematic random sampling method was applied to select the study participants, and pretested and structured questionnaires were used to collect data. The collected data were coded, entered, cleaned, and analyzed using SPSS version 20.0 software. Binary logistic regression analyses with a confidence interval of 95% were used to select determinant factors. Statistically significant factors were identified using the adjusted odds ratio (AOR). Statistical significance was determined at *p* value <0.05. Binary and multivariable logistic regression analyses were applied to see the association of the variables at a *p* < 0.05.

**Results:**

In this study, the overall proportion of neonatal sepsis was (60.2%) 95% CI (56, 68)]. Birth asphyxia [AOR = 2.04; 95%CI (1.07, 3.93)], maternal age of 15–19 [AOR = 2.00; 95% CI (1.81, 11.93)], duration of labor greater or equal to 24 hours [AOR = 3.00; 95% CI (2.67, 14.21)], history of oxygen administration [AOR = 2.37; 95% CI (1.18, 4.75)], neonatal age of greater or equal to seven days [AOR = 4.0595% CI (1.07, 3.93**)**, and home delivery [AOR = 5.00; 95% CI (2.34, 18.92)] were the predictor variables for neonatal sepsis.

**Conclusion:**

In this study, neonatal sepsis was high. Birth asphyxia, intranasal oxygen administration, age of the mother, home delivery, and duration of labor were associated with neonatal sepsis.

## 1. Introduction

A neonate, often known as a newborn infant, is a baby who is born within the first 28 days of life [1]. Neonatal sepsis (NS) is a systemic inflammatory response syndrome as a result of suspected or in the presence of or proven infection in a neonate within the first four weeks [2]. Neonatal sepsis is classified as early-onset sepsis (if the onset of clinical features occurs between birth and 7 days) or late-onset neonatal sepsis (LONS) if the onset of clinical features occurs between 8 and 28 days after birth [3].

Globally, sepsis is one of the major causes of mortality and morbidity in neonates up to now [4]. The World Health Organization (WHO) estimates that 1 million deaths per year (10% of all under-five mortality) are because of neonatal sepsis and that 42% of these deaths occur in the first week of life [5].

There are great differences in neonatal care between low- and high-income countries. In high-income countries, the main issue is the increasing number of extremely premature infants with high nosocomial infection rates because of the multidrug resistant organisms in intensive care units. Similarly, hospital-acquired infections are also a major concern in low-income countries, but the major problem is the high proportion of home deliveries in unclean environments predisposing to sepsis [5].

Based on the reports in Ethiopia, the pooled prevalence of neonatal sepsis was 45% with a reported range of neonatal sepsis from 17% to 78% [6]. Null parity, duration of labor >18 h after rupture of membranes, gestational age of 32–37 weeks, neonates who require resuscitation at birth, neonatal age, place of delivery, lack of training of health workers on neonatal resuscitation and infection prevention practices, history of urinary tract infections during the index pregnancy, and frequency of per-vaginal examination are some of the determinant factors of neonatal sepsis [7, 8].

Even if the effect of neonatal sepsis is a major public health problem in resource-limited countries like Ethiopia, a scanty finding on the magnitude and factors associated with neonatal sepsis is available. Therefore, this study aimed to assess the prevalence and associated factors of neonatal sepsis among neonates admitted to the Neonatal Intensive Care Unit (NICU) in Hawzen Primary Hospital.

## 2. Materials and Methods

### 2.1. Study Design, Area, and Period

An institution-based cross-sectional study design was conducted in Hawzen Primary Hospital, Tigray, Northern Ethiopia, in January-March/2020.

### 2.2. Source and Study Population and Inclusion and Exclusion Criteria

All neonates admitted to Hawzen Primary Hospital were the source population, while all selected neonates in Hawzen Primary Hospital during the study period were the study population. The inclusion criteria were neonates who were admitted to the NICU and/or pediatrics ward of Hawzen Primary Hospital. Neonates with sepsis admitted two or more times during the study period were considered to be excluded to avoid double counting.

### 2.3. Sample Size and Sampling Procedure

The sample size was calculated using a single population formula (*n* = Z2 p (1 − p)/d2), with a confidence level specified at 95%, where *z* is the normal standard distribution set at 1.96, and a tolerable margin of error (d) at 5%, considering the nonresponse rate of 10% and the prevalence of neonatal sepsis estimated to be 78% [9] taken from a previous similar study done in Ethiopia. Finally, the sample size was 290. Study participants were selected by using a systematic random sampling method to get the final sample size.

### 2.4. Study Variables

The outcome variable was neonatal sepsis (yes/no) sociodemographic factors of the neonate and mother, obstetric factors of the mother, birth-related factors of the neonate, and medical factors were the independent variables of this study.

### 2.5. Data Collection Tool, Measurements, and Quality Management

The tool was developed from different types of literature. The questionnaire was first developed in the English language and translated into Tigrigna (local language), and it was retranslated back again to the English language to check for any inconsistencies. A pilot was done out of the study area in 5% of the sample to identify any misunderstanding of questionnaire and to set the time needed to complete the interview. Then, the necessary correction was made accordingly before the beginning of data collection, and one-day training was given to three data collectors and one supervisor. Data were collected from the mother or caregiver of the neonates by using the interviewer-administered structured Tigrigna version questionnaire that contains detailed questions including all the variables of the study. The data collectors also reviewed the neonate's medical record to check the final clinical diagnosis of neonatal sepsis prior to the data collection.

### 2.6. Data Processing and Analysis

The collected data were coded and entered using Epi info version 7.2.1.0 and then exported to SPSS version 25 for the purpose of analysis. After that, descriptive statistics was used to analyze accordingly and summarized using percentage and frequency. Both binary and multivariable logistic regression analyses were used to determine factors associated with neonatal sepsis. In order to include very important variables, variables with *p* < 0.2 in the bivariable analysis were entered into multivariable logistic regression. The factors associated with neonatal sepsis were determined by calculating the adjusted odds ratio (AOR) and crude odds ratio (COR) with a confidence interval (CI) of 95%. *p* value <0.05 was used to determine statistical significance. Finally, text and tables were used to present the analyzed data accordingly. The goodness of fit for the final logistic model was tested using the Hosmer and Lemeshow test at a value of >0.05.

Operational definition; neonatal sepsis: Neonates presented with any one of the systemic manifestations of danger signs: drowsy or unconscious, not feeding well, convulsions, movement only when stimulated or no movement at all, grunting severe chest in-drawing, raised temperature >38°C, hypothermia <35.5°C, fast breathing (60 breaths per min), central cyanosis or could be severe jaundice, severe abdominal. Distension or localizing signs of infection were diagnosed as having neonatal sepsis. Late onset of sepsis: if sepsis is occurring between 8 and 28 days of age. Early onset of sepsis: if sepsis is occurring from birth to 7 days of age.

## 3. Results

### 3.1. Neonatal Characteristics

A total of 287 mother-neonate pairs were included in Hawzen primary hospitals in the Eastern zone of Tigray, Ethiopia, making the response rate 98.6%. The median age of neonates was 3 days with an interquartile range of 4 days. Among all participants, more than half of 149 (52%) were female neonates. Nearly two-thirds of neonates were born at hospital 180 (62.7%). Similarly, nearly three-fourths of 201(70.1%) neonates were term. Low birth weight was reported among 52% of neonates (Table 1).

### 3.2. Mothers Sociodemographic and Economic Characteristics

Nearly one-third of neonate mothers 78 (27.1%) were in the age range of 25 to 29 years. About 259(90.2%) of neonates' mothers were married. About 104(36.6) of neonates' mothers were able to read and write. More than half of 166 (57.8%) were from the rural Table 2.

### 3.3. Medical Procedures Related to Neonatal Health Care Services

About 23(8.1%) % of neonates had a history of central venous catheterization. About 53(18.5%) of the neonates had a history of oxygen administration. About 12(4.2%) of the neonates had a history of nasogastric tube insertion. None of the neonates were on mechanical ventilation (Table 3).

### 3.4. Mothers' Medical and Obstetric Conditions

In this study, nearly half of the mothers 141(49.1%) were nulliparous women. About 253(88.1%) of the mothers had antenatal care follow-up. About 14(4.9) of the mothers were positive for HIV. About 227(79%) of the mothers were delivered spontaneously through the vagina. About 32(11.1) of the mothers had a history of foul-smelling liquor. Nearly one-third 95(33.1) of the mothers had a history of premature rupture of the membrane (Table 4).

### 3.5. Clinical Characteristics of Neonates

In this study, birth asphyxia was 62(21.6%) and birth injury was 56(19.5%) (Table 5).

### 3.6. The Proportion of Neonatal Sepsis

**Figure 1:**
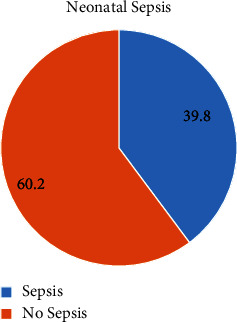
The proportion of neonatal sepsis among neonates admitted in Hawzen primary hospitals, Tigray, Northern Ethiopia, 2020.

In this study, the overall magnitude of neonatal sepsis was 60.1% 95% CI (58, 68) (Figure 1).

### 3.7. Factors Associated with Neonatal Sepsis

Maternal age category, residency, age of the neonate, sex of neonate, place of birth, gestational age, birth weight, frequency of PV examination, history of NG tube insertion, maternal age, maternal marital status, duration of labor, history of PROM, history of foul smelling liquor, history of IV catheterization, history of neonatal resuscitation, birth asphyxia, place of delivery, and parity fulfilled the variable screening criteria (*p* value <0.2) and entered into multivariable logistic regression analysis. Consequently, the age of the neonate, maternal age, place of delivery, resuscitation at birth, and laboring hour were significantly associated with the risk of onset of neonatal sepsis at multivariable with less than 0.05 *p* values. Accordingly, duration of labor showed a significant association with the risk of the onset of neonatal sepsis. The odds of having neonatal sepsis among women laboring for 24 hours or greater were 3 times higher as compared to mothers laboring 6 hours or less [AOR = 3.00; 95% CI (2.67, 14.21)].

According to this study, neonates who had birth asphyxia were 2 times more likely to develop neonatal sepsis than those who had no birth asphyxia [AOR = 2.04; 95% CI (1.07, 3.93)]. This study showed that maternal age was significantly associated with neonatal sepsis. Neonates born from mothers whose ages are 15–19 had 2 times higher odds of developing sepsis as compared to neonates from mothers whose age is 35 years or older [AOR = 2.00; 95% CI (1.81, 11.93)].

Similarly, those neonates who had a history of oxygen administration were 2.37 more likely to develop neonatal sepsis compared to those who had no history of oxygen administration [AOR = 2.37; 95% CI (1.18, 4.75)].

This study also identified significant associations between neonatal age in days and neonatal sepsis. The odds of developing neonatal sepsis among neonates whose ages were greater than or equal to seven days were 4.05 times higher as compared to neonates whose ages were less than or equal to seven days [AOR = 4.05; 95% CI (1.07, 3.93)].

Finally, place of delivery was also significantly associated with the risk of onset of neonatal sepsis. The odds of developing neonatal sepsis were 5 times higher as compared to those who deliver at the hospital [AOR = 5.00; 95% CI (2.34, 18.92)] (Table 6).

## 4. Discussion

Neonatal sepsis contributes substantially to neonatal mortality and morbidity and is a major global public health challenge, especially in developing countries [10]. In this study, the overall magnitude of the neonatal sepsis was 60.1% 95% CI (58, 68)].

This finding is in line with the study done in Gondor, Ethiopia (64.8%). Similar results might be the result of similarities in the research period in which the study was conducted. The reason for the similar result might be due to the same study period. This study is however higher than studies done in Ethiopia (34%) [7], Nigeria (34%) [11], Tanzania (31.4%) [12], Uganda (11%) [13], and India (32%) [14]. This difference might have been attributed to the sample size difference, sociodemographic factors, cultural factors, and the health professional difference in diagnostic knowledge, skill, and modalities to confirm neonatal sepsis. On the contrary, this result was lower as compared to studies done in Bishoftu, Ethiopia (72.2%) [15], Shashemene, Ethiopia (77.9%) [9], Woldia and Dessie, Ethiopia (79.4%) [16], and Arbaminch General Hospital, Ethiopia (78.9) [17]. The reason could be these study sites are referral hospitals, most of the time receiving referred neonates with complications as well as referred mothers for delivery with complications. In addition, the difference in the study area, sociodemographic factors, economic factors, and culture might be the reason.

In this study, duration of labor showed a significant association with the risk of the onset of neonatal sepsis. The odds of having neonatal sepsis among women laboring for 24 hours or greater were 3 times higher as compared to mothers laboring 6 hours or less. This is in agreement with a study from Ethiopia [7].

This research also discovered a link between those neonates who had a history of oxygen administration and neonatal sepsis neonates who had a history of oxygen administration were 2.37 more likely to develop neonatal sepsis compared to those who had no history of oxygen administration. This finding is in line with studies carried out in Shashemene, Ethiopia [9].

Maternal age was also significantly associated with neonatal sepsis. Neonates born from mothers whose age is 15–19 had 2 times higher odds of developing sepsis as compared to neonates from mothers whose age is 35 years or older. This finding is in agreement with studies conducted in Bangladesh and Tanzania [12, 18]. However, studies conducted in Ethiopia showed that neonates from mothers whose age was from 30 to 34 years were 81% less likely to develop neonatal sepsis as compared to those neonates from mothers older than 35 years [8]. The discrepancy might be differences in the study sample size and methodological difference.

Depending on this study, neonatal sepsis was linked with birth asphyxia. Neonates who had birth asphyxia were 2 times more likely to develop neonatal sepsis than those who had no birth asphyxia. This result is in line with a study from Shashemene, Ethiopia, and Indonesia [9, 19]. Neonatal asphyxia facilitates systemic infections. This is due to inhibited leukocyte activity because it requires energy (ATP) for cytoskeletal microfilament contractions. The state of hypoxia will also inhibit the microbicidal activity of polymorphonuclear cells [20].

The place of birth was also found to be statistically significant with neonatal sepsis. The place of delivery was also significantly associated with the risk of onset of neonatal sepsis. The odds of developing neonatal sepsis were 5 times higher as compared to those who delivered at the hospital. This is in parallel with studies from Nigeria [11]. The reason could be most home deliveries are carried out in unclean environments which predisposes to sepsis [5].

Finally, this study indicated that neonatal age in days has significant associations with neonatal sepsis. The odds of developing neonatal sepsis among neonates whose ages were greater than or equal to seven days were 4.05 times higher as compared to neonates whose ages were less than or equal to seven days. This finding is in line with the findings from a study in Ghana, and Shashemene, Ethiopia [9, 21]. Nosocomial and community-acquired neonatal infections occur after 3 days of life. Consequently, this will affect the prevalence of late-onset neonatal sepsis [22].

## 5. Conclusion

In this study, neonatal sepsis was high. Birth asphyxia, history of intranasal oxygen administration, age of the mother 15–19 years old, home delivery, age of the neonate greater or equal to 7 days, and duration of labor greater or equal to 24 hours were associated with neonatal sepsis. So, health professionals might work on the risk factors to minimize neonatal sepsis.

### 5.1. Limitation

There might be an overestimation of neonatal sepsis due to the diagnosis was made clinically. In addition, this study was conducted in one primary hospital, so it may not represent to the general population.

## Figures and Tables

**Table 1:** Socio-demographic characteristics of neonates admitted to neonatal intensive care units (NICUs) of Hawzen primary hospitals, Tigray, Northern Ethiopia, 2020.

Variable		Frequency (%)
Sex	Male	138 (48%)
	Female	149 (52%)

Age	0–7 days	234 (81%)
	8–28 days	53 (19%)

Birth weight	≥4000 g	14 (4.8%)
	2500–3499 g	124 (43.2%)
	1500–2499 g	120 (41.9%)
	<1500 g	29 (10.1%)

Place of delivery	Health center	86 (30%)
	Hospital	180 (62.7%)
	Home	21 (7.3%)

Gestational age	37–42 weeks	201 (70.1%)
	<37 weeks	86 (29.9%)

**Table 2:** Socio-demographic characteristics of mothers of neonates admitted to NICU in Hawzen primary hospitals, Tigray, Northern Ethiopia, 2019.

Variable		Frequency (%)
Residence	Urban	121 (42.2%)
	Rural	166 (57.8%)

Maternal age	15–19 years	25 (8.8%)
	20–24 years	68 (23.8%)
	25–29 years	78 (27.1%)
	30–34 years	72 (25%)
	≥35 years	44 (15.3%)

Marital status	Married	259 (90.2%)
	Unmarried	28 (10.8%)

Maternal educational level	Unable to read and write	29 (10.2%)
	Able to read and write	104 (36.6%)
	Primary education	57 (19.8%)
	Secondary education	66 (22.9%)
	Diploma and above	31 (10.8%)

Occupation	Housewife	234 (81.6%)
	Government employee	26 (9%)
	NGO/private	20 (6.9%)
	Others	7 (2.5%)

**Table 3:** Medical procedures done for neonates who were admitted to NICU in Hawzen primary hospitals, Tigray, Northern Ethiopia, 2020.

Variables		Frequency (%)
Central venous catheterization	Yes	23 (8.1%)
	No	264 (91.9%)

Nasogastric tube insertion	Yes	12 (4.2%)
	No	272 (95.8%)

Oxygen administration	Yes	53 (18.5%)
	No	234 (81.5%)

Endotracheal tube intubation	Yes	9 (3.1%)
	No	278 (96.9%)

Mechanical ventilation	No	287 (100%)

**Table 4:** Medical and obstetrical characteristics of mothers of neonates admitted to NICU in Hawzen primary hospitals, Tigray, Northern Ethiopia, 2020.

Variables		Frequency (%)
Parity	I	141 (49.1%)
	II	82 (28.5%)
	III and more	64 (22.4%)

Antenatal care	Yes	253 (88.1%)
	No	34 (11.9%)

HIV test result	Positive	14 (4.9%)
	Negative	264 (92%)
	Unknown	9 (3.1%)

VDRL/RPR status	Reactive	26 (9%)
	Nonreactive	254 (88.6%)
	Unknown	7 (2.4%)

Mode of delivery	Spontaneous vaginal delivery	227 (79%)
	Cesarean section	20 (6.9%)
	Instrumental	40 (14.1%)

Duration of labor	<6 hours	102 (35.5%)
	6–12 hours	62 (21.6%)
	12–24 hours	78 (27.2%)
	>24 hours	45 (15.7%)

Frequency of PV exam during labor	<3	121 (42.1%)
	>3 times	166 (57.9%)

History of foul-smelling liquor	Yes	32 (11.1%)
	No	255 (88.9%)

History of PROM	Yes	95 (33.1%)
	No	192 (66.9%)

History of high-grade fever during pregnancy	Yes	20 (7%)
	No	267 (93%)

VDRL: venereal disease research laboratory test; RPR: rapid plasma regain test; PV: pelvic examination; PROM: premature rupture of membrane.

**Table 5:** Clinical characteristics of neonates who were admitted to Hawzen primary hospital, Tigray, Northern Ethiopia, 2020.

Variable		Frequency (%)
Birth injury	Yes	56 (19.5%)
	No	231 (80.4%)

Birth asphyxia	Yes	62 (21.6%)
	No	224 (78%)

Gross congenital malformation	Yes	50 (17.4%)
	No	237 (82.6%)

Cry at birth	Yes	200 (69.7%)
	No	87 (30.3%)

**Table 6:** Bivariable and multivariable analysis of factors associated with neonatal sepsis among neonates admitted at Hawzen primary hospitals, Tigray, Northern Ethiopia, 2020.

Variable		Neonatal sepsis *N* (%)		COR (95% CI)	AOR (95% CI)
		Yes	No		
Maternal age category	15–19 years	15 (5.3)	10 (3.5)	1.31 (1.23, 9.39)	**2.00 (1.81, 11.93)** ^∗^
	20–24 yrs.	38 (13.3)	30 (10.5)	0.45 (0.18, 2.3)	0.62 (0.23, 2.61)
	25–29 yrs.	60 (20.9)	18 (6.2)	0.69 (0.30, 6.91)	1
	30–34 yrs.	40 (19.3)	32 (5.7)	0.34 (0.21, 2.9)	0.21 (0.18, 2.01)
	≥35 yrs.	28 (9.8)	16 (5.5)	1	1

Residence	Urban	55 (19.2)	66 (23)	1	1
	Rural	100 (34.8)	66 (23)	0.54 (0.22, 11.39)	0.9 (0.1, 10.9)

Marital status	Married	150 (52.3)	109 (37.9)	1	1
	Not married	8 (2.8)	20 (8)	0.75 (0.32, 3.67)	0.34 (0.11, 9.12)

Parity	I	100 (34.8)	41 (14.3)	0.23 (0.18.2.35)	0.98 (0.32, 6.74)
	II	40 (13.9)	42 (14.6)	0.79 (0.34, 3.80)	0.81 (0.67, 11.89)
	III	40 (13.9)	24 (8.5)	1	1

Duration of labor	<6 hours	50 (17.4)	52 (18.1)	1	1
	6–12 hours	28 (9.8)	34 (11.8)	0.35 (0.12, 3.25)	0.98 (0.35, 9.00)
	12–24 hours	48 (16.7)	30 (10.5)	1.1 (0.99, 13.24)	0.21 (0.19, 14.13)
	>24 hours	35 (12.2)	10 (3.5)	2.12 (1.43, 10.91)	**3.00 (2.67, 14.21)** ^∗^

Sex of neonate	Male	80 (27.8)	58 (20.2)	1	1
	Female	49 (17.2)	100 (34.8)	0.20 (0.15, 6.81)	0.98 (0.51, 3.45)

Gestational age	<37 weeks	48 (16.7)	38 (13.2)	0.93 (0.24, 8.90)	0.34 (0.21, 8.13)
	37–42 weeks	150 (52.3)	51 (17.8)	1	1

Frequency of PV examination	≤3 times	80 (27.8)	41 (14.3)	1	1
	>3 times	100 (34.8)	66 (23.1)	1.76 (0.67, 12.85)	0.24 (0.17, 6.98)

History of birth asphyxia	Yes	17 (5.9)	45 (15.6)	5.94 (3.14, 11.11)	**2.04 (1.07, 3.93)** ^∗^
	No	155 (54.0)	69 (2.4)	1	1

VDRL test result	Reactive	18 (6.2)	8 (2.8)	1	1
	Nonreactive	150 (52.3)	104 (36.3)	1.23 (0.90, 3.760	0.87 (0.45, 5.12)
	Unknown	5 (1.7)	2 (0.7)	0.98 (0.31, 11.34)	1.2 (0.31, 8.76)

History of oxygen administration	Yes	30 (10.4)	23 (8.1)	4.07 (2.44, 6.79)	**2.37 (1.18, 4.75)** ^∗^
	No	200 (69.8)	34 (11.7)	1	1

History of NG tube insertion	Yes	7 (2.4)	5 (1.8)	1.2 (0.9, 12.53)	5.34 (0.98, 15.35)
	No	200 (69.7)	72 (26.1)	1	1

History IV catheterization	Yes	10 (3.5)	13 (4.6)	2.98 (1.38, 19.01)	6.78 (0.67, 18.95)
	No	173 (60.2)	91 (31.7)	1	1

History of neonatal resuscitation	Yes				
	No			1	1

Birth weight	<1500 g	20 (7)	9 (3.1)	0.67 (0.18, 10.00)	2.19 (0.98, 20.21)
	1500–2499 g	70 (25.8)	50 (17.4)	3.41 (0.56, 9.12)	6.11 (0.98, 17.34)
	≥2500 g	90 (31.4)	48 (16.6)	1	1

Foul-smelling vaginal discharge	Yes	20 (6.9)	12 (4.2)	11.4 (9.48, 27.35)	5.98 (0.17, 13.25)
	No	134 (46.6)	121 (42.3)	1	1

Neonatal age in days	≤7 days	160 (55.7)	74 (25.3)	1	1
	>7 days	27 (9.4)	26 (9.0)	1.91 (1.08, 3.37)	**4.05 (1.07, 3.93)** ^∗^

History of PROM	Yes	50 (17.4)	45 (15.7)	0.67 (0.31, 17.34)	0.98 (0.36, 5.77)
	No	100 (34.8)	92 (32.1)	1	1

Place of delivery	Home	12 (4.2)	9 (3.1)	1.98 (1.1, 12.32)	**5.00 (2.34, 18.92)** ^∗^
	Health center	50 (17.5)	36 (12.5)	0.43 (0.171, 4.45)	0.87 (0.52, 9.87)
	Hospital	14 (4.8)	7 (2.5)	1	1

^∗^ *p* value <0.05.

## Data Availability

The datasets used and/or analyzed during the current study are available from the corresponding author on reasonable request.
